# Proprioceptive and olfactory deficits in individuals with Parkinson disease and mild cognitive impairment

**DOI:** 10.1007/s13760-023-02420-w

**Published:** 2023-11-14

**Authors:** Kuan-yi Li, Kristen A. Pickett, Hsuan-wei Fu, Rou-shayn Chen

**Affiliations:** 1 Department of Occupational Therapy and Graduate Institute of Behavioral Sciences, Chang Gung University, Tao-Yuan, Taiwan; 2 Healthy Aging Research Center, Chang Gung University, Taoyuan, Taiwan; 3 Division of Movement Disorders, Department of Neurology, Chang Gung Memorial Hospital at Linkou, Taoyuan, Taiwan; 4 Department of Kinesiology, University of Wisconsin-Madison, Madison, WI USA; 5 Occupational Therapy Program, University of Wisconsin-Madison, Madison, WI USA; 6 Department of Rehabilitation, Kuang Tien General Hospital, Taichung, Taiwan

**Keywords:** Perceptual deficits, Position sense, Smell, Non-motor symptoms, Parkinson’s disease, Dementia

## Abstract

**Background:**

Individuals with neurodegenerative diseases such as Parkinson disease (PD) and Alzheimer’s (AD) disease often present with perceptual impairments at an early clinical stage. Therefore, early identification and quantification of these impairments could facilitate diagnosis and early intervention.

**Objectives:**

This study aimed to compare proprioceptive and olfactory sensitivities in individuals diagnosed with PD and mild cognitive impairment (MCI).

**Methods:**

Proprioception in the forearm and olfactory function were measured in neurotypical older adults, individuals with PD, and individuals with MCI. Position and passive motion senses were assessed using a passive motion apparatus. The traditional Chinese version of the University of Pennsylvania smell identification test (UPSIT-TC) and the smell threshold test (STT) were used to identify and discriminate smell, respectively.

**Results:**

Position sense threshold between the groups differed significantly (*p* < 0.001), with the PD (*p* < 0.001) and MCI (*p* = 0.004) groups showing significantly higher than the control group. The control group had significantly higher mean UPSIT-TC scores than the PD (*p* < 0.001) and MCI (*p* = 0.006) groups. The control group had a significantly lower mean STT threshold than the PD and MCI groups (*p* < 0.001 and *p* = 0.008, respectively). UPSIT-TC scores significantly correlated with disease progression in PD (r = − 0.50, *p* = 0.008) and MCI (r = 0.44, *p* = 0.04).

**Conclusions:**

Proprioceptive and olfactory sensitivities were reduced in individuals with PD and MCI, and these deficits were related to disease severity. These findings support previous findings indicating that perceptual loss may be a potential biomarker for diagnosing and monitoring disease progression in individuals with neurodegenerative diseases.

## Introduction

Parkinson's disease (PD) and Alzheimer's disease (AD) are the two most common neurodegenerative disorders affecting older adults. They may share common neuropathological mechanisms, as they exhibit overlapping clinical and pathological features [1]. However, the precise neural mechanisms remain unclear. Both diseases are characterized by protein misfolding and aggregation in the brain [2] and share similar early-stage symptoms, including olfactory impairments, sleep disturbances, and depression [3, 4]. Individuals with neurodegenerative diseases, such as PD and AD, often experience perceptual impairments at an early stage. Hence, early identification and quantification of these impairments could facilitate early interventions. Moreover, perceptual assessments offer an accessible, non-invasive, and cost-effective clinical tool compared with other laboratory tests or neuroimaging examinations. They can also provide valuable insights into neurological diseases.

PD is a common neurodegenerative disease resulting from nigrostriatal dopaminergic neuron loss in the basal ganglia [5]. People with PD (PwPD) experience various perceptual deficits, including olfactory dysfunction and proprioceptive deficits [6, 7]. PwPD demonstrate difficulties in detecting limb position and passive motion required for accurate movements [8] and exhibit decreased olfactory sensitivity in identifying and discriminating smell [9]. Evidence from muscle vibration studies have suggested that the proprioception deficits observed in PwPD are more likely due to sensorimotor integration errors instead of peripheral impairments [10]. Similarly, other neurological disorders, such as dementia and mild cognitive impairment (MCI), have been associated with perceptual loss.

MCI is the decline of cognitive function beyond normal aging and is characterized by memory and thinking difficulties without functional limitations [11]. People with MCI (PwMCI) have a higher risk of developing dementia [11], and early cognitive intervention could stimulate neural reorganization and potentially reduce cognitive decline [12]. People with dementia exhibit prominent olfactory impairment [13], whereas PwMCI have measurable olfactory decline, although less severe than those with dementia [14]. This finding may illustrate the extent to which disease progression is related to perceptual loss in individuals with neurodegenerative diseases.

Compared with laboratory tests or neuroimaging examinations, perceptual assessments offer an easy-to-administer, non-invasive, and affordable clinical tool, which may offer valuable insights into neurological diseases. Therefore, this study aimed to systematically examine proprioceptive and olfactory deficits in PwPD and PwMCI and compare these findings to those of neurotypical older adults. Specifically, we measured proprioceptive thresholds in the forearm and assessed olfactory function in healthy older adults, PwPD, and PwMCI. By quantifying proprioceptive and olfactory sensitivity, we aimed to examine the degree of perceptual impairment in people with early-stage neurodegenerative conditions and examine the potential use of perceptual assessments as a screening battery for neurodegenerative disorders.

## Methods

### Participants

All participants (including healthy, PwPD, and PwMCI) were required to meet the same set of inclusion and exclusion criteria to address cognitive impairment, depression, peripheral nerve disorders, history that might affect olfactory function, and orthopedic or neurological conditions that might affect proprioception. Inclusion criteria were as follows: 1) Mini-mental state examination (MMSE) score ≥ 24 [15]; 2) Beck depression inventory score ≤ 20 [16]; 3) no signs or symptoms of peripheral nerve disorders, such as peripheral neuropathy; 4) no history of smoking; and 5) no medical history, including chronic rhinosinusitis, nasal allergy, history of nasal surgery, or head trauma, which might affect olfactory function, as determined by an otorhinolaryngologist. Exclusion criteria included: 1) diagnosis of any other neurological disorders, such as stroke; 2) any medical history of injury to the extremities that may affect proprioceptive sensitivity, such as shoulder dislocation or joint replacement; 3) diabetes (owing to its association with peripheral neuropathy); 4) inability to follow instructions and focus on the procedure for 30 min; and 5) upper respiratory tract infection. The inclusion criterion for age was set between 50 and 65 years for healthy participants.

Additional inclusion criteria for PwPD involved those: 1) aged 50–65 years; 2) with idiopathic PD diagnosed by a movement disorder neurologist; and 3) with Hoehn & Yahr stage classification < III. PwPD with more tremor-related symptoms were excluded because involuntary movement might interfere with their ability to detect arm position and motion. PwPD underwent testing in the “on” medication state for Unified Parkinson Disease Rating Scale (UPDRS) and the entire experiment. Daily medication doses were standardized using established formulas [17]: 100 mg standard levodopa = 125 mg sustained-release levodopa, 1.5 mg pramipexole, 6 mg ropinirole, 10 mg bromocriptine, or 1 mg pergolide.

Additional inclusion criteria for PwMCI included: 1) age ≥ 60 years; 2) Montreal Cognitive Assessment (MoCA) score ≤ 26, adjusted for age and education [18, 19]; 3) no difficulties in basic activities of daily (ADL) performance as assessed using the Barthel Index [20]; and 4) no diagnosis of dementia by a physician.

PwPD were recruited from the Neurology outpatient clinic at the Chang Gung Memorial Hospital. PwMCI and healthy participants were recruited from the community in Northern Taiwan. All participants provided oral and written informed consent and received a copy of the consent form before the test. The study was approved by the local institutional review board (IRB no: 201200603B0D001).

### Proprioceptive testing apparatus

**Fig. 1:**
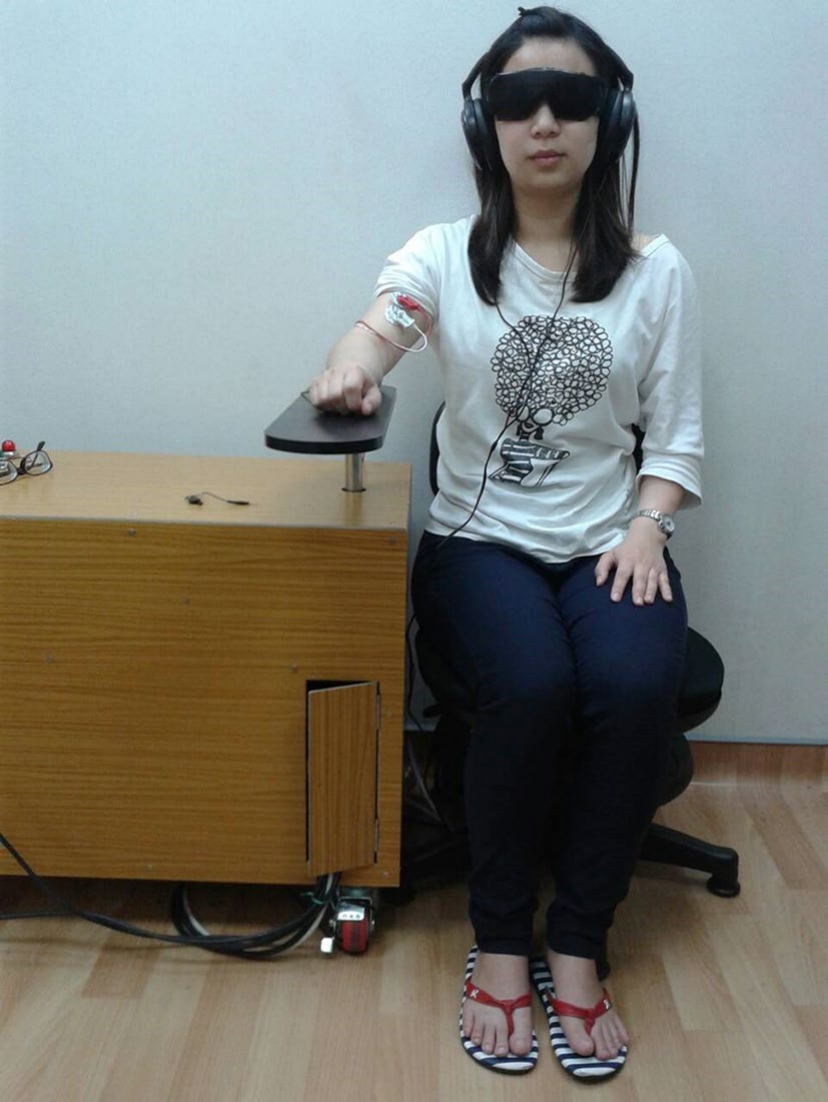
Proprioceptive apparatus experimental setup. Participants sat on a chair with their vision and hearing occluded. Each participant’s forearm was placed on the splint, with the elbow at 90º. EMG electrodes were positioned over the biceps brachii to monitor muscle activity during the trials

The apparatus designed to quantify proprioceptive sensitivity comprised a rectangular metal splint (60 × 9 cm) and torque motor (DELTA servo motor, 400 w). The motor is attached to the splint at the pivot end and generates torque to rotate the displacement end of the splint in the transverse plane (Fig. 1). The pivot end of the splint is supported by an adjustable shaft that allows height adjustment to accommodate each participant. The torque motor can generate angular speeds between 0.0002º/s and 300º/s.

It is controlled by a computer application with a graphical user interface, which enables experimental protocol implementation. Two handheld triggers enable online control of the apparatus during individual trials. Pressing the trigger stops the motor, enabling the measurement of movement duration and displacement. Splint rotation occurs with little vibration and noise to prevent vibration and audible cueing.

### Procedure

Before testing, each participant underwent an initial screening that focused on the inclusion and exclusion criteria. Individuals completed several intake measures and assessments, including a standard demographic form, handedness assessment using the Edinburgh Handedness Inventory [21], and a medical history form. A member of the study team evaluated disease progression and severity. PwPD completed the Movement Disorder Society’s (MDS-UPDRS) [22], whereas PwMCI completed the MOCA [18].

#### Measuring proprioceptive sensitivity

The height of the chair and apparatus were adjusted based on each participant’s seated height. Each participant placed their forearm on the splint, aligning their elbow with the pivot point on the testing apparatus. Participants were instructed to relax their arm on the rectangular splint while their limb was placed in the starting position with their shoulder slightly abducted and their elbow flexed to 90º. A handheld goniometer was used to ensure consistency of the starting position. Participants wore goggles and headphones with pink noise to obscure visual and auditory cues and distractors during testing. Each trial began with the researcher tapping the participant’s shoulder to indicate the beginning of a new trial. The tactile cue was accompanied by the verbal command “concentrate now,” which repeatedly reminded the individual to focus on the task.

Each participant completed two proprioceptive testing procedures, including passive motion sense (PMS) and limb position sense (LPS). Each trial of the PMS task involved the participant comparing two angular velocities. For the PMS task, the comparison velocities ranged between 1.58º/s and 2.70º/s, with step increments of 0.15º/s. Eight different comparison velocities were used: 1.58º/s, 1.73º/s, 1.88º/s, 2.03º/s, 2.18º/s, 2.33º/s, 2.58º/s, and 2.70º/s. Each trial comprised a standard velocity of 1.50º/s paired with one of the eight comparison velocities. Each velocity was presented for 2 s before switching to the other velocity. The interval between the two velocities was 500 ms. Within a single trial, the two velocities to be discriminated always moved in the same direction (flexion or extension). After each trial, the participant verbally indicated which velocity was faster.

LPS trials followed a similar pattern. The comparison displacements for LPS ranged between 8.8º and 9.92º, with step increments of 0.16º. Eight different comparison displacements were used: 8.8º, 8.96º, 9.12º, 9.28º, 9.44º, 9.60º, 9.76º, and 9.92º. Each trial comprised a standard displacement of 10º paired with one of the eight comparison displacements. The movement direction for each pair of stimuli was always away from the body. The inter-stimulus interval between the standard and comparison angular displacements was approximately 1 s. For the first stimulus, each participant's arm was passively moved to the desired angular displacement at a constant velocity of 2º/s and then returned to the starting position. After a 1-s interval, the participant's arm moved again to the second desired angular displacement with the same constant velocity. At the end of each trial, participants had to indicate which angular displacement was further away from their body.

In both proprioceptive testing procedures, the standard and comparison stimuli were presented randomly. If a participant reported losing focus or needing to repeat a trial, the trial would be repeated only once. A standard forced-choice paradigm was used throughout the testing procedure; therefore, participants could not respond with “I do not know” or “They were the same,” rather, the individual was asked to select the trial that met the specific task instructions. The experimenter recorded each participant’s verbal response at the end of each trial.

LPS testing was always conducted first, as our pilot testing indicated that participants had more difficulty with the LPS task than the PMS task. Before beginning each testing paradigm, three practice trials were administered to confirm that participants understood the experimental procedure. Standard surface EMG with a sampling rate of 1000 Hz was used to monitor myoelectric activities of the biceps to ensure participants remained passive and did not generate any movement during the test. Trials exhibiting visible electromyography (EMG) activity were excluded and repeated once without being counted towards the 72 LPS and 72 PMS trials. The more affected arm was examined in PwPD, whereas the dominant arm was examined in PwMCI and the control group.

To gain further insights into the participants' strategies and explore their approaches during the trials, we conducted an "exit interview" after the task.

#### Olfactory function

The traditional Chinese version of the University of Pennsylvania smell identification test (UPSIT-TC), a modified version of the original UPSIT [23], was used to measure each participant’s odor identification function (Sensonics, Inc. Hadden Heights, NJ). The validity and reliability of UPSIT-TC have been established [24]. The UPSIT-TC comprises four 10-odor booklets. Each “scratch-and-sniff” odor is embedded in a microcapsule and covered in a brown rectangular area at the bottom of each page. Participants were instructed to scratch the brown area with a pencil tip in a standardized manner and choose the correct answer from four choices. The total score for the UPSIT-TC ranges from 0 to 40, with 40 indicating no errors made.

The smell threshold test (STT) (Sensonics, Inc., Hadden Heights, NJ) was used to assess smell discrimination. The STT has been examined for its correlation with other olfactory tests and test–retest reliability [23, 25]. Two sniff bottles in a quasi-random sequence were provided on the threshold response forms. The participants had to judge which odorant in a given pair was stronger. The total score on the STT ranges from − 10 to − 2, with higher negative values indicating better performance.

### Data analysis and statistics

Proprioceptive sensitivity data were analyzed using customized MATLAB algorithms. The percentage of correct responses was computed for each stimulus intensity, and a psychometric function was generated. The just noticeable difference thresholds (JNDTs) for LPS and PMS were defined as the intensity at which participants achieved a 75% correct response rate. The UPSIT-TC score was determined by summing the correct responses. The mean STT threshold was determined using a fixed staircase procedure to measure the discrimination threshold, defined as the mean of the last four out of seven staircase reversal points.

A one-way analysis of covariance (ANCOVA) was used to examine group differences between the PD, MCI, and control groups regarding proprioceptive and olfactory sensitivities after controlling for age. Post-hoc analyses using the least significant difference tests were performed when justified. The Pearson correlation coefficient was used to examine the relationships between proprioceptive and olfactory sensitivities, disease duration, disease progression, medication level, and age. Statistical significance was set at *p* < 0.05 for ANCOVA and *p* < 0.016 for post-hoc analyses (adjusted for multiple comparisons).

## Results

### Characteristics of participants

This study included 32 individuals with idiopathic PD, 22 with MCI, and 30 healthy elderly adults. Age significantly differed between the groups (*p* < 0.001), with the MCI group being significantly older than the other two groups (both *p* < 0.001). All participants were right-handed except two controls and two PwPD. Table 1 provides detailed demographic characteristics.

**Table 1:** Participant demographic characteristics

	PD (*n* = 30)	MCI (*n* = 22)	Control (*n* = 30)
Age, years (mean ± SD)	62.3 ± 5.4	76.64 ± 7.80	62.33 ± 3.41
Sex (Male/Female)	22/8	18/4	19/11
Handedness (Right/Left)	28/2	22/0	28/2
MMSE (mean ± SD)	28.0 ± 1.7	24.77 ± 3.61	29.07 ± 1.17
More affected side (Right/Left)	17/13		
Disease duration, years (mean ± SD)	4.1 ± 2.3		
Hoehn & Yahr stage	1.9 ± 0.5		
UPDRS total score (mean ± SD)	37.2 ± 15.1		
UPDRS motor score (mean ± SD)	23.7 ± 9.2		
Levodopa equivalent dose, mg/day, (mean ± SD)^a^	766.7 ± 663.2		

*MMSE* Mini-Mental State Examination, *UPDRS* Unified Parkinson’s Disease Rating Scale

^a^100 mg standard levodopa equals 125 mg sustained release levodopa, 1.5 mg pramipexole, 6 mg ropinirole, 10 mg bromocriptine, or 1 mg pergolide (Fahn, 1999)

### Proprioceptive sensitivity

The mean JNDTs for LPS were 0.68º ± 0.20º (mean ± SD), 0.97º ± 0.12º, and 1.03º ± 0.37º in the control, PD, and MCI groups, respectively. A main effect of the group was found (F_2,78_ = 10.00; *p* < 0.001). Post-hoc analysis revealed that the JNDTs for LPS in the PD and MCI groups were significantly higher than in the control group (*p* < 0.001, CI: 0.19–0.52; and *p* = 0.004, CI: 0.11–0.53, respectively).

**Fig. 2:**
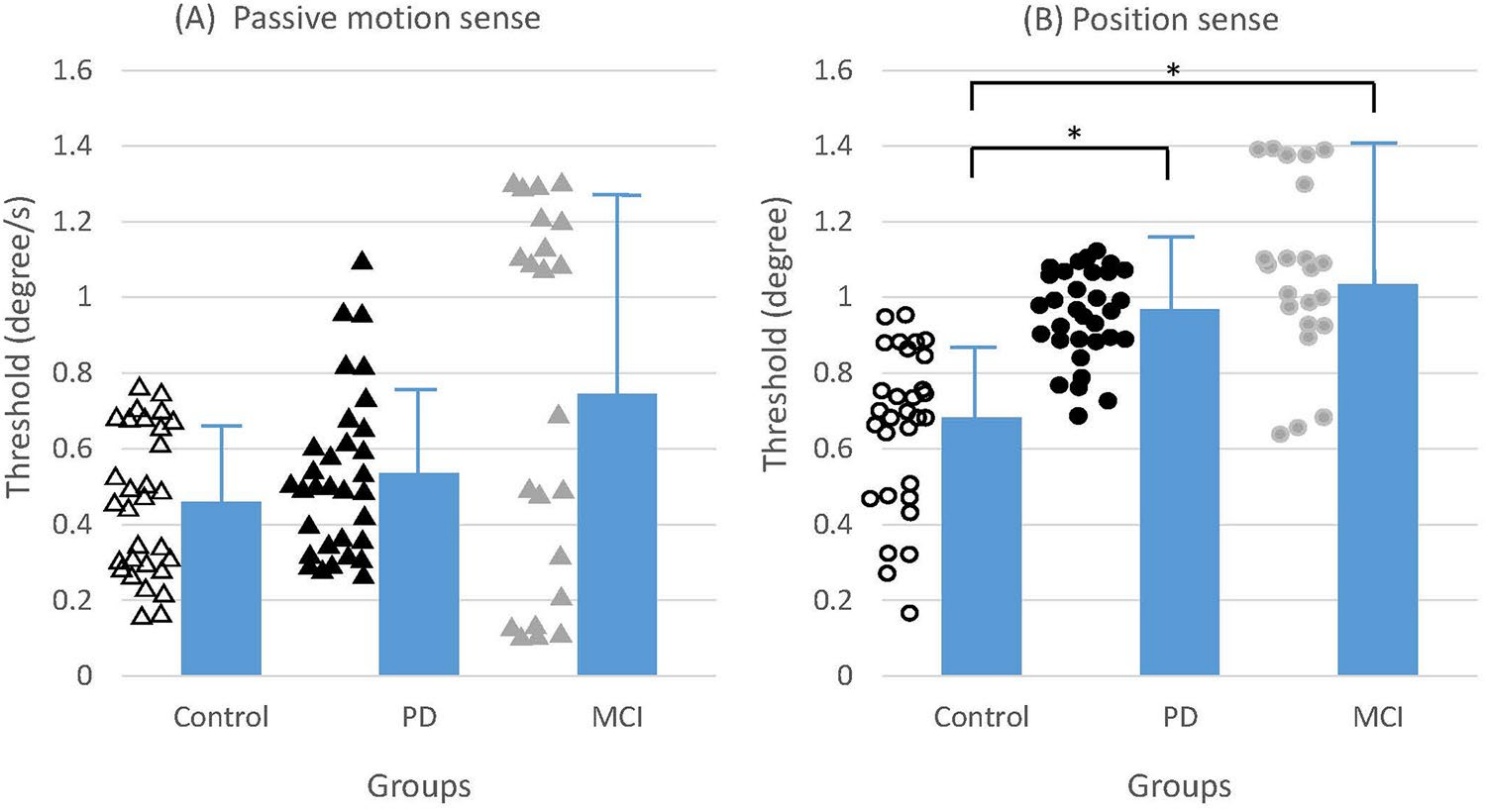
Mean JNDTs for **a** passive motion and **b** position sense in the control, PD, and MCI groups. The asterisk represents a significant difference between groups (*p* < 0.05). Each triangle represents the threshold of one participant

The mean JNDTs for PMS were 0.46º/s ± 0.19º/s, 0.54º/s ± 0.22º/s, and 0.74º ± 0.51º in the control, PD, and MCI groups, respectively. No significant group effect was found. On average, participants experienced 3–5 trials that displayed EMG activity or resulted in attention loss, requiring repetition. In such instances, the experimenter would remind the participant of the experimental instructions or provide a break if needed. Figure 2 presents proprioceptive testing results.

### Olfactory sensitivity

The mean UPSIT-TC scores were 28.00 ± 3.62, 17.31 ± 5.21, and 19.32 ± 7.38 in the control, PD, and MCI groups, respectively. A significant main effect of the group was found (F_2, 78_ = 17.97; *p* < 0.001). The post-hoc analysis revealed that the control group had significantly higher mean UPSIT-TC scores than the PD and MCI groups (*p* < 0.001, CI: 7.62–15.21; and *p* = 0.006, CI: 2.04–11.90, respectively).

**Fig. 3:**
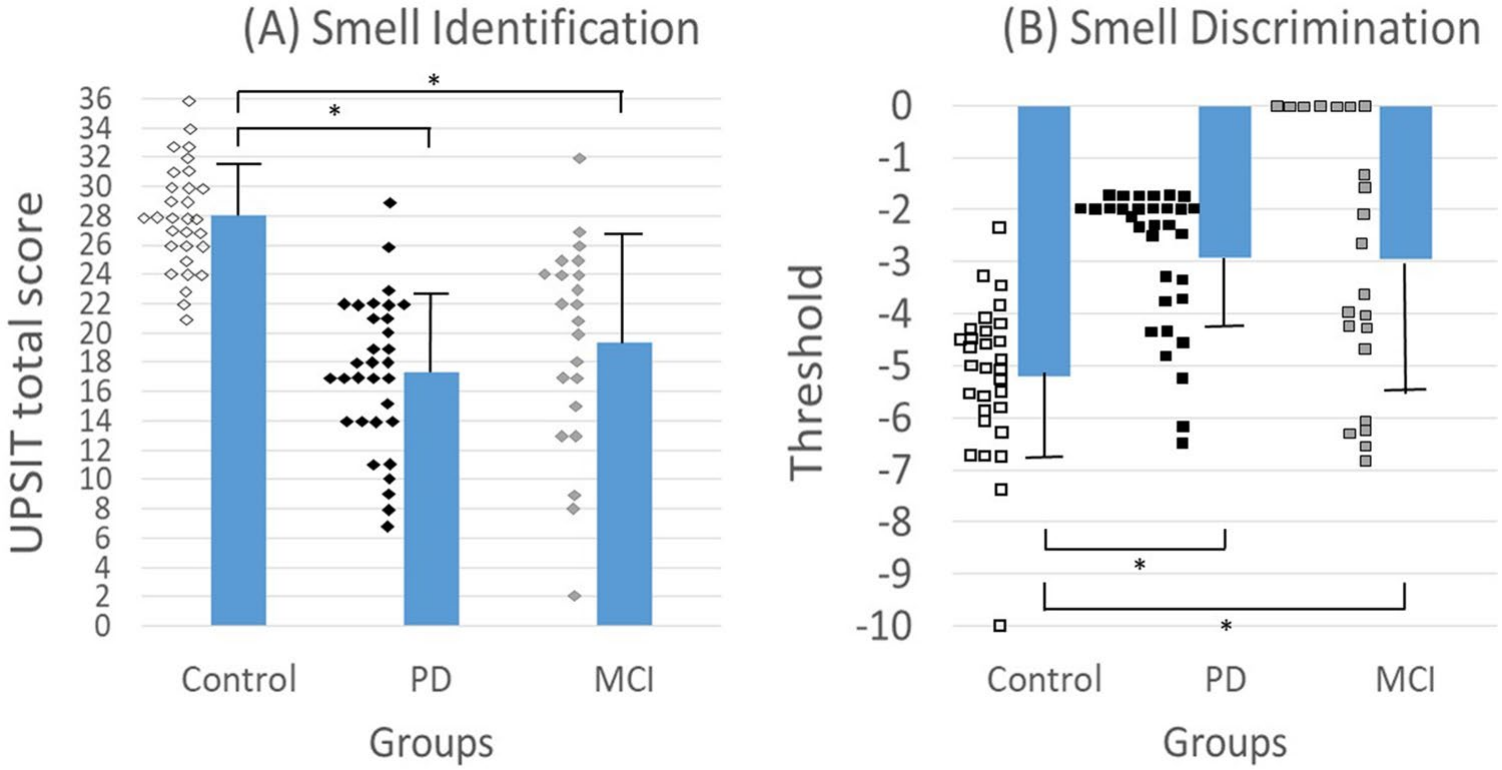
Mean **a** UPSIT total scores and **b** discrimination thresholds for smell identification and discrimination in the control, PD, and MCI groups. The asterisk represents a significant difference between groups (*p* < 0.05). Greater negative values for the smell discrimination threshold represent greater sensitivity to smell discrimination. Each square represents the threshold of one participant. Abbreviations: UPSIT, University of Pennsylvania Smell Identification Test

The mean STT scores were − 5.22 ± 1.46, − 2.91 ± 1.34, and − 2.95 ± 2.54 in the control, PD, and MCI groups, respectively. A significant main effect of group was indicated (F_2, 78_ = 8.67; *p* < 0.001), with post-hoc analysis indicating that the control group had significantly lower scores than the PD and MCI groups (*p* < 0.001, CI: − 3.80– − 1.30; and *p* = 0.008, CI: − 3.85–− 0.59, respectively). Figure 3 presents the olfactory testing results.

### Correlation analysis

A group-level correlation analysis revealed that the UPSIT-TC score was negatively correlated with disease duration (r = − 0.52, *p* = 0.002) and UPDRS total score (r = − 0.50, *p* = 0.008) in PwPD. Furthermore, the UPSIT-TC score was positively correlated with the MoCA score (r = 0.44, *p* = 0.04) in the MCI group. Age did not significantly correlate with sensory function across the three groups. Table 2 presents the detailed results of correlational analyses.

**Table 2:** Pearson correlations between proprioceptive and olfactory sensitivity and age across the three groups

Measures	JNDT for passive motion sense		JNDT for limb position sense		Smell identification		Smell discrimination	
	r	*p* values	r	*p* values	r	*p* values	r	*p* values
*Healthy group*								
Age	− 0.11	0.95	− 0.24	0.20	0.05	0.79	− 0.08	0.67
*PD group*								
Age	0.22	0.23	0.20	0.28	− 0.14	0.44	0.12	0.51
Disease duration	< 0.001	1.00	− 0.15	0.41	− 0.52	0.002**	0.06	0.76
UPDRS motor	− 0.03	0.88	− 0.09	0.65	− 0.30	0.13	0.24	0.22
UPDRS total	− 0.08	0.68	− 0.19	0.34	− 0.50	0.008**	0.18	0.36
Levodopa equivalent dose	− 0.14	0.43	− 0.01	0.96	− 0.13	0.49	0.31	0.09
*MCI group*								
Age	0.39	0.07	0.17	0.46	− 0.19	0.39	0.05	0.82
MoCA	− 0.43	0.05	0.06	0.78	0.44	0.04*	− 0.40	0.07

*JNDT* Just noticeable difference threshold, *UPDRS* Unified Parkinson’s Disease Rating Scale, *PD* Parkinson’s disease, *MCI* mild cognitive impairment, *MoCA* Montreal Cognitive Assessment.

*: *p*-value < 0.05; **: *p*-value < 0.01

## Discussion

This study examined whether PwPD and PwMCI experience proprioceptive and olfactory deficits compared with neurotypical older adults. Further, the relationships between proprioception, olfaction, and disease progression were examined for each patient population. The study's primary results indicate that individuals in both neurodegenerative populations exhibited impairments in limb position discrimination and smell identification and discrimination. Furthermore, a corresponding increase in perceptual decline was observed with disease progression. These findings improve our understanding of perceptual changes in early-stage PD and MCI and may provide insights into how perceptual testing can monitor disease progression.

The typical onset age for PD and MCI differs, making it impractical to fully match the age for all three groups. PD typically begins around 50 [26], and as individuals age, PD is often accompanied by comorbidities such as osteoarthritis and diabetes mellitus. These comorbidities can limit the participants' ability to perform certain LPS and PMS tests. Furthermore, PwMCI have an average age of 70–75 years [27]. Considering the onset age of PD and MCI without comorbidities, which could exclude or interfere with the results, we set the age range for the control and PD groups as 50–65 years and 60 years for the MCI group. We used age as the covariate for data analysis to control for the potential effect of age.

### Proprioceptive sensitivity is reduced in PwPD and PwMCI

Both groups with neurodegenerative diseases exhibited proprioceptive deficits compared with neurotypical older adults. The ability to discriminate limb position was impaired in PwPD and PwMCI, whereas the ability to discriminate passive motion was not impaired. The mean JNDTs for LPS in the PD and MCI groups were 142% and 151% higher than that of the control group. The mean JNDTs for PMS in the PD and MCI groups were 117% and 162% higher than that of the control group. To our knowledge, this study is the first to report impaired position sense in PwMCI.

MCI is primarily associated with cognitive impairment and is often considered a potential precursor to the development of AD [28]. A connection exists between MCI and abnormalities in sensory-perceptual processing and motor deficits [29]. PwMCI experience auditory and speech processing impairments, particularly affecting the brain areas involved in these functions [29]. Moreover, disruptions in fine movement control have been observed among individuals with MCI and AD [30, 31]. These findings highlight sensory-perceptual and motor deficits in PwMCI. This study builds on the previous literature by examining proprioceptive sensitivity in PwMCI and showing decreased sensitivity in discriminating displacement and passive motion of the forearm compared with older adults without MCI.

Many studies have documented impairment in detecting position and motion senses in PwPD [7, 32, 33]. However, our findings demonstrate that proprioceptive deficits are not limited to detecting movement but also extend to differentiating displacement at the forearm for PwPD in the earlier stages of disease progression. This suggests that reduced proprioceptive sensitivity may have potential implications.

These proprioceptive deficits are associated with PD motor symptoms such as bradykinesia and hypometria [34, 35]. Klockgether & Dichgans (1994) reported that PwPD usually overestimate the movement range in the more affected limb during simultaneous bilateral arm movements, leading to hypometric movement and undershooting of the targets. They also rely more on external cues, such as visual information, owing to their proprioceptive deficit [36]. PwPD have prolonged movement times, increased end-point position errors, and greater variability in the extent and direction of hand motion than those without PD [34, 35]. Therefore, altered kinesthesia in PD may cause an inappropriate estimation of hand motion; however, the errors in hand motion probably reflect a deficit in sensorimotor integration rather than kinesthesia only.

For participant selection, we recruited individuals with mild PD based on a modified Hoehn and Yahr scale < III [37], followed by the UPDRS assessment. However, we observed a discrepancy between the UPDRS score and Hoehn and Yahr Scale for defining early-stage PD. Skorvanek et al. (2017) reported differences in MDS-UPDRS scores based on the Hoehn and Yahr Scale and disease duration. They found that over 30% of PwPD were classified as severity level 2 or higher on the MDS-UPDRS section III at Hoehn and Yahr Scales 1 and 2. Specifically, the average scores for part III (on phase) were 14.4 ± 7.8 and 28.8 ± 12.3 for Hoehn and Yahr Scales 1 and 2, respectively [38]. This is consistent with the distribution of UPDRS scores in our participants. In our study, most PwPD had UPDRS motor scores below 30; however, seven (23%) had scores greater than 30.

### Potential central neural network of proprioception

Proprioception provides non-visually guided self-awareness of the position and motion of the body and limb segments [34]. The impaired proprioceptive sense in PwPD resulted from errors from the central origin rather than those in the peripheral afferents (including Golgi tendon organs, joint receptors, or muscle spindle fibers) [7, 34]. Furthermore, PwPD have other perceptual impairments besides proprioception, including tactile discrimination, and weight perception [39, 40]. Additionally, brain imaging studies have suggested abnormal sensory processing in subcortical and cortical areas in PwPD [41]. These findings support the theory that the basal ganglia play a fundamental role as a sensory analyzer for processing somatosensory signals [41, 42], and the intact cerebro-basal ganglia loop is largely involved in processing proprioceptive signals [42].

Intact proprioception is an essential component of fine and gross motor movement, as the brain must accurately understand the position of the limbs in space to properly control movement. Therefore, the motor cortex heavily relies on proprioceptive information as part of the sensory-motor integration cycle [34]. If proprioceptive signals are altered because of dysfunction in the basal ganglia, such as in PwPD, the erroneously located limb cannot be accurately or efficiently controlled. Our findings indicate that this sensory-motor loop may be disrupted early during the disease progression.

Individuals with PD and AD often exhibit similar psychological symptoms (such as depression and sleep disturbance); however, the existing literature is limited in fully addressing the potential dysfunction of the basal ganglia and proprioceptive impairments in PwMCI. Kazee et al. (1995) reported that AD is linked to pathological lesions in the substantia nigra. However, their study did not find a correlation between the level of lesion and disease progression [43]. Furthermore, Perl et al. (1998) argued that AD and PD might share a common neuropathological mechanism because of the overlap in clinical and pathological features [1]. However, the overlap in the neural mechanism between these two disorders remains unclear. Our study suggests that the underlying disease mechanism and/or affected neurophysiology of PwPD and PwMCI may involve a common pathway associated with perceptual deficits.

### Olfactory sensitivity is reduced in PwPD and PwMCI

In our olfactory testing, PwPD and PwMCI showed deficits compared with neurotypical older adults in both assessment paradigms. Additionally, smell identification was significantly correlated with disease duration and progression in the PD group and with cognition in the MCI group.

Similar to proprioception, we discovered that the PD and MCI groups exhibited impaired olfactory function compared with older adults without a neurodegenerative diagnosis. The deficit was evident in detecting and discriminating olfactory stimuli. Previous studies have documented olfactory impairment in PwPD and PwMCI using assessment batteries focused on smell identification and discrimination and odor memory recognition [14, 44]. Similar to our findings, these deficits were observed in the early stages of the disease [13], and olfactory functional loss increases as AD progresses [45]. Similar patterns have been observed in PwPD and those with similar neurodegenerative syndromes (Parkinsonism). These findings have led researchers to consider olfactory testing as a possible differential diagnostic tool [45]. People with progressive supranuclear palsy have more sensitive olfactory function than those with idiopathic PD, whereas individuals with corticobasal degeneration retain intact olfaction [46]. Based on these differential findings, olfactory function has been considered a potential biomarker for neurodegenerative diseases at the preclinical stage.

### Potential central neural network of olfaction

The olfactory pathway originates from the olfactory bulb in the periphery and connects to the anterior olfactory nucleus, piriform cortex, and anterior amygdala in the central region of the brain [47]. Increased blood flow has been observed in the right orbitofrontal cortex and the connection between the inferior frontal lobe and occipital lobe during smelling [48]. Disruption of this pathway at various points can result in impaired olfactory function, as observed in individuals with AD and PD, considering the different underlying pathology between the two disorders [45].

In individuals with AD, neurofibrillary tangles and amyloid plaques have been observed in the olfactory bulbs and olfactory pathways to the cortex, including the anterior amygdala and peri-amygdaloid cortex [47]. These peripheral and central olfactory neuropathologies result in olfactory deficits. In contrast, Lewy bodies and Lewy neurites resulting from the accumulation of α-synuclein were observed in the olfactory bulbs in PwPD. This accumulation leads to disrupted neural transmission, axonal degeneration, and eventual neuron death [49, 50]. In the cortex, similar pathological changes have been observed in the olfactory processing areas, including the olfactory tubercle, frontal piriform cortex, and temporal piriform cortex in PwPD [51]**.** However, whether the olfactory deficits resulted from the pathological changes in the olfactory pathway or dopamine loss from the substantia nigra remain inconclusive. Based on these findings, examining the perceptual impairments and the underlying neurophysiology to fully understand the progression of neurodegenerative disease is important. Psychophysical and imaging-based findings should be considered when establishing biomarkers. However, perceptual decline may be an important early sign in identifying high-risk populations [45].

### Age did not significantly correlate with sensory function

In proprioception and olfactory function assessments, we did not observe a significant correlation between age and sensory sensitivity within each group. Sensory functions tend to decline with age; however, our findings did not align with this expectation. There are two possible explanations for these results.

First, olfactory impairment can start as early as age 50 and progressively worsen with age [52]. However, the prevalence of olfactory dysfunction significantly rises in individuals aged 60 and older, with men being more susceptible than women [53]. This suggests that age alone may not be the sole determinant of olfactory dysfunction in our study. Similarly, a correlation between aging and a decline in joint position sense and movement detection threshold has been demonstrated [54, 55]. However, the effect of age on proprioceptive sensitivity remains inconclusive [56], as regular physical activity may help mitigate proprioception decline [54, 57]. This could explain why we did not observe a significant correlation between age and proprioceptive function within each group.

Second, the pathological effects of the diseases in the PD and MCI groups had a more substantial effect on kinesthetic and olfactory impairments than aging. This may account for the lack of a significant correlation between age and sensory function within each group. These findings suggest that age alone may not be the primary factor influencing sensory function within our groups. Other factors, such as disease pathology and regular physical activity, might have played a significant role in determining sensory impairments.

### Correlations with clinical scores and medication

The correlational analysis results indicated that only smell identification demonstrated a significant correlation with disease progression in both PD and MCI groups. In the PD group, smell identification also exhibited a correlation with disease duration. These findings align with prior research, indicating that both PD and AD manifest severe olfactory identification impairment in the early stages of the conditions, with olfaction appearing to progressively worsen over time [13, 58]. The exact mechanisms underlying olfactory dysfunction in PD and AD are not yet fully understood. However, it is suggested that both peripheral and central olfactory pathways may contribute to this issue. On the other hand, the relationship between clinical scores, medication, and proprioceptive sensitivity in the PD group remains inconclusive, as suggested by previous studies [7, 33, 59]. In relation to medication, a study by Jobst et al. (1997) found that levodopa did not improve proprioceptive deficits in blindfolded PD participants. They did not show increased sensitivity in perceiving differences in movement amplitudes while taking levodopa. Furthermore, other studies indicate that dopamine replacement therapy (DRT) may have a negative impact on proprioceptive function in PD [60, 61]. However, Li et al. (2010) reported that individuals with mild to moderate PD experience improvements in haptic and proprioceptive function with the use of levodopa, suggesting positive effects on perceptual function. The discrepancies in findings may be attributed to the variations in the assessment tasks employed to measure proprioceptive function in previous studies. In contrast to previous studies that relied on motor performance measures, our study utilized a passive motion apparatus along with established psychophysical methods to assess discrimination thresholds. These thresholds serve as reliable indicators of perceptual sensitivity.

### Participants mainly relied on proprioception for discriminating angular displacements and velocities instead of a time estimation strategy

In our study, participants primarily relied on proprioception for discriminating angular displacements and velocities, guided by the comprehensive instructions provided before the task and the feedback from the exit interview. All participants met the inclusion criteria, having a Mini-Mental State Examination score greater than 24, which indicated no signs of dementia and ensured their ability to comprehend the experiment.

To conduct the PMS task, we presented each velocity for 2 s before transitioning to the next velocity, ensuring consistency in stimulus duration. The comparison velocities ranged from 1.58º/s to 2.70º/s, with an increment of 0.15º/s. Eight different comparison velocities were utilized. In each trial, we paired a standard velocity of 1.50º/s with one of the eight comparison velocities, separated by a 500-ms interval. LPS trials followed a similar pattern. The comparison displacements for LPS ranged between 8.8° and 9.92°, with step increments of 0.16°. Eight different comparison displacements were used. In each trial, a standard displacement of 10º was paired with one of these eight comparison displacements. Participants' arms were passively moved to the desired angular displacement at a constant velocity of 2º/s during the trials. Consequently, the duration for each displacement ranged between 4.4 to 5 s, posing a challenge in discerning slight differences in time duration.

Based on the findings from the "exit interview" after the task, most participants reported that perceiving the displacement was easier than counting the time duration in the LPS task. While we cannot completely rule out the possibility that some participants may have used the counting time strategy to discriminate between two angular displacements, evidence suggests that participants primarily relied on sensing the displacement based on the comprehensive instructions provided before the task and the feedback obtained from the exit interview.

Similar to the LPS task, most participants expressed that it was easier to perceive and discriminate between velocities when the velocity was higher. Conversely, participants found it easier to sense the displacement for discrimination when the velocity was lower. The order of comparison velocities was randomly presented, making it unpredictable for participants. Despite this, most participants stated that it was easier to simply sense the velocity for the experimental task. The above evidence indicates that participants primarily relied on perceiving the velocities based on the comprehensive instructions provided before the task and the feedback from the exit interview.

In summary, considering the constant time duration in the passive motion sense task and the minimal time differences in the LPS task, participants primarily relied on sensing displacements and velocities rather than cognitive processes. Individuals with Parkinson's disease may have impairments in time estimation; however, these limitations are unlikely to significantly affect the overall findings of the study.

### Limitations

This study had some limitations. First, the mean ages of the groups were not matched. We opted to enroll participants into all three groups simultaneously rather than using age-matching, as the effect of age on proprioception remains inconclusive for proprioceptive sensitivity [56]. A previous study indicated no significant difference in position sense at the hip joint between young and old participants [31, 57]. Moreover, the typical onset age for PD and MCI differs, posing a challenge in matching the age between the two groups. We accounted for age as a covariate in our group comparisons; however, this approach may not eliminate the potential influence of age on the differences in sensory thresholds between the groups. Our results demonstrated that PwMCI had 162% and 151% higher thresholds for proprioception than the control group for the PMS and LPS, respectively. Therefore, the difference in proprioceptive sensitivity observed between the MCI and control groups was beyond any potential effect of aging. The mean UPSIT total score for smell identification was still lower than normal, even after adjusting the score for Taiwanese cultural differences [62]. Second, the ratio of males to females was not equivalent in the PD and control groups. We cannot fully rule out the potential effect of sex on proprioceptive sensitivity; however, evidence to demonstrate that a sexual difference in proprioceptive sensitivity remains unclear [63, 64]. Third, we only measured sensitivity in perceiving displacement and passive motion at the elbow joint in the transverse plane of one arm. Therefore, the findings may not be generalizable to other limb segments. Finally, tactile and pressure cues were minimized; however, they could not be fully removed during testing because of the apparatus design and the individual placement on the apparatus. However, these cues were similar for all participants. Therefore, tactile and pressure cues were likely not the primary stimuli for discriminating limb position and passive motion.

## Conclusion

This study is the first to report impaired position sense in PwMCI. Proprioceptive sensitivity and olfactory function were impaired in PwPD and PwMCI. Furthermore, smell identification was significantly correlated with disease duration and progression in the PD group and with cognition in the MCI group. These findings support previous findings indicating that perceptual loss may be a potential biomarker for diagnosing and monitoring disease progression in individuals with neurodegenerative diseases.

## Data Availability

The datasets generated during and/or analyzed during the current study are available from the corresponding author on reasonable request.
